# Impact of underweight status on mortality in sepsis patients: a meta-analysis

**DOI:** 10.3389/fmed.2025.1549709

**Published:** 2025-02-06

**Authors:** Jiaan Chen, Fan Zhang, Li Liang, Xuming Pan, Jiancheng Zhang, Guangjun Jin

**Affiliations:** ^1^Department of Clinical Medicine, The Second Clinical Medical College, Zhejiang Chinese Medical University, Hangzhou, China; ^2^Department of Emergency, The Second Affiliated Hospital of Zhejiang Chinese Medical University, Hangzhou, China

**Keywords:** 1-year mortality, in-hospital mortality, meta-analysis, sepsis, underweight

## Abstract

**Objective:**

The evidence regarding the impact of underweight status on clinical outcomes in patients with sepsis are still scarce and controversial. We aimed at conducting a meta-analysis to evaluate the potential associations between underweight and the mortality rate among sepsis patients.

**Methods:**

A comprehensive electronic search was performed in PubMed, Cochrane Library, Embase, and Web of Science databases. Odds ratios (ORs) or mean differences and 95% confidence intervals (CIs) were calculated using RevMan 5.3.

**Results:**

A total of 58,348 patients (normal weight group: 49,084 patients; underweight group: 9,264 patients) from 23 studies were included in this meta-analysis. The results indicated that the in-hospital mortality (OR, 1.28; 95% CI, 1.21, 1.35; heterogeneity: *I*^2^ = 21%, *P* = 0.21), 28-day mortality (OR, 1.54; 95% CI, 1.26, 1.88; heterogeneity: *I*^2^ = 74%, *P* < 0.0001) and 1-year mortality (OR, 1.78; 95% CI, 1.58, 2.00; heterogeneity: *I*^2^ = 41%, *P* = 0.17) of underweight patients were significantly higher than those of normal weight patients. However, there was no significant difference in length of hospital stay or intensive care unit length of stay between underweight patients and normal-weight patients.

**Conclusion:**

Underweight is associated with increased mortality in patients with sepsis. Physicians should pay more attention to the management of underweight sepsis patients.

**Systematic review registration:**

https://www.crd.york.ac.uk/PROSPERO/display_record.php?RecordID=631417, identifier CRD42025631417.

## 1 Introduction

Sepsis is a life-threatening syndrome caused by the body’s dysfunctional immune response to a serious systemic infection, often involving multiple organs and systems (1, 2). Sepsis and septic shock are important global health problems, and sepsis is the leading cause of death in intensive care units (3). In the United States, sepsis is now more common than heart attacks and strokes (3). According to statistics, there are about 50 million cases of sepsis worldwide each year, resulting in more than 10 million deaths annually, accounting for about 20% of global deaths (1, 2). Although much progress has been made in the pathobiology and epidemiology of sepsis in recent years, the high mortality rate of the disease remains a concern for clinicians (3). Therefore, it is critical to identify prognostic factors for sepsis to help clinicians identify high-risk patients early and make more informed medical decisions.

Previous evidence suggests that body mass index (BMI) may be an important prognostic factor for death in patients with sepsis (4–6). A meta-analysis of 15 studies by Bai et al. (4) showed that overweight and obesity (BMI = 25.0–39.9 kg/m^2^) were associated with reduced mortality in patients with sepsis or septic shock. A recent multicenter cohort study also confirmed that obese patients had better in-hospital survival and better functional outcomes at discharge compared to non-obese patients (5). In Asian countries, fewer people are overweight and obese than in Western countries. In general, Asians have a lower BMI than Europeans (7). Therefore, it is necessary to study the effects of low BMI on patients with sepsis, especially in Asian countries. However, the relationship between underweight and the prognosis of sepsis remains controversial. A retrospective study by Li et al. (8) found that underweight significantly increased the risk of death in patients with sepsis. However, Sakr et al. (9) showed no significant difference in in-hospital mortality between patients with sepsis in the low BMI and normal BMI groups.

To overcome the current dilemma of conflicting results, we conducted a comprehensive review of published evidence and meta-analysis to determine whether being underweight increases the risk of death in patients with sepsis. These results may have important clinical implications for understanding the prognosis of patients with underweight sepsis and improving clinical decision making.

## 2 Materials and methods

### 2.1 Search strategy

This study was conducted in accordance with the Preferred Reporting Items for Systematic Reviews and Meta-Analyses (PRISMA). The study was registered in the PROSPERO database.

Two investigators (Jiaan Chen and Fan Zhang) independently performed a systematic literature review using the Web of Science, PubMed, Embase, and Cochrane Library databases to identify studies published before 29 November 2024. The detailed search strategy is presented in Table 1. In addition, we checked the reference lists of the eligible articles to identify other potential studies. No language restrictions were applied during the search process.

**TABLE 1:** Electronic search strategy.

Database	Search term (published up to 29 November 2024)	Number
PubMed	(underweight[Title/Abstract] OR low body mass index[Title/Abstract] OR low BMI[Title/Abstract] OR low body weight[Title/Abstract]) AND (sepsis[Title/Abstract] OR Pyemia [Title/Abstract] OR Pyohemia[Title/Abstract] OR septicemia[Title/Abstract] OR septic[Title/Abstract])	161
Embase	(low body mass index OR low BMI OR underweight OR low body weight).ab,kw,ti. AND (sepsis OR Pyemia OR Pyohemia OR septicemia OR septic).ab,kw,ti.	44
Cochrane Library Trials	((low body mass index OR low BMI OR underweight OR low body weight):ti,ab,kw) AND ((sepsis OR Pyemia OR Pyohemia OR septicemia OR septic):ti,ab,kw)	49
Web of Science	(TS = (low body mass index OR low BMI OR underweight OR low body weight)) AND (TS = (sepsis OR Pyemia OR Pyohemia OR septicemia OR septic))	512

### 2.2 Study selection

Studies meeting the following criteria were analyzed: (1) Patient: patients admitted to the intensive care unit (ICU) and treated for sepsis, severe sepsis, or septic shock; (2) Intervention: patients who are underweight (BMI < 18.5 kg/m^2^); (3) Comparison: patients of normal weight (BMI = 18.5–24.9 kg/m^2^) (according to the World Health Organization definition) (10); (4) Outcomes: the primary outcome was in-hospital mortality. Secondary outcomes included 28-day mortality, 90-day mortality, 1-year mortality, ICU length of stay and hospital stay; and (5) Study type: cohort studies and case-control studies.

The exclusion criteria were as follows: case reports, duplicate literature, letters, studies available only as abstracts, unpublished manuscripts, and animal studies.

### 2.3 Data extraction

Two investigators (Jiaan Chen and Fan Zhang) independently extracted data, which included author name, study design, country, year of publication, period of study, study population (age, sample size, and diagnosis), and outcome information (in-hospital mortality, 28-day mortality, 90-day mortality, 1-year mortality, ICU length of stay, and hospital stay). When data of interest were unavailable, the corresponding author was contacted to obtain the missing data.

### 2.4 Quality assessment

The quality assessment was conducted independently by two authors (Jiaan Chen and Fan Zhang) using the Newcastle-Ottawa Scale (NOS), which assigns a score on a 9-point scale. A score of ≥7 indicates high quality, and scores of 5–6 indicate moderate quality. Any discrepancies were resolved by a third author (Guangjun Jin).

### 2.5 Statistical analysis

The meta-analysis was performed using the Review Manager software (version 5.3). Odds ratios (ORs) with corresponding 95% confidence intervals (CIs) were calculated for categorical outcome variables and mean difference (MD) for continuous outcome variables. Heterogeneity among studies was assessed by using the I-squared (*I*^2^) statistic. A random-effects model was employed if *I*^2^ > 50%; otherwise, a fixed-effects model would be used (11). To explore the robustness of the outcomes, we adopted the one-study exclusion method to evaluate the impact of each study on the total effect size. The subgroup analysis was conducted based on age and the region where the study was conducted. Publication bias was assessed using Egger’s test and funnel plot when the number of studies was greater than 10. Statistical significance was established at *P*-value of less than 0.05.

## 3 Results

### 3.1 Literature retrieval

**FIGURE 1:**
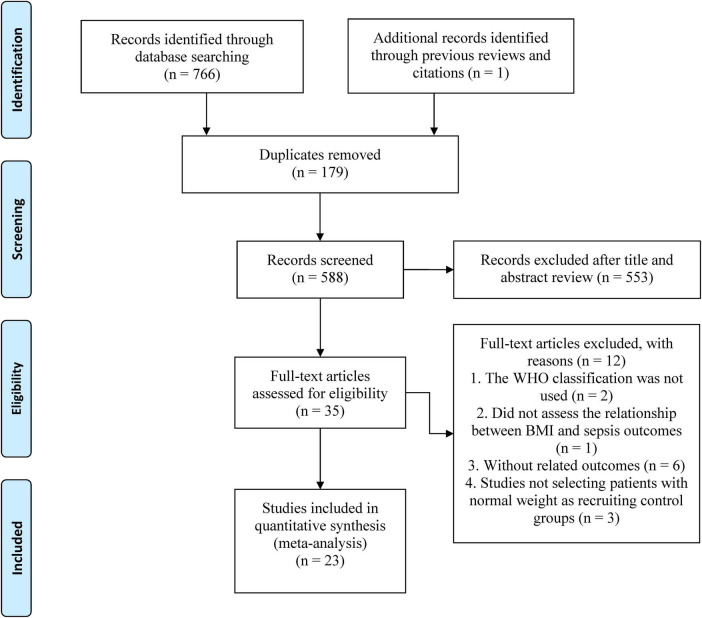
The PRISMA flowchart.

Our literature search identified 767 records, of which 179 were duplicates. After reviewing titles and abstracts, 553 studies were excluded, and the full texts of the remaining 35 studies were evaluated. Finally, 23 studies (1–3, 7–9, 12–28) were included in our meta-analysis (Figure 1).

### 3.2 Study characteristics and quality assessment

The detailed characteristics of the 23 studies (1–3, 7–9, 12–28) were described in Table 2. The studies were published between 2008 and 2024 and included 58,348 patients (normal weight group: 49,084 patients; underweight group: 9,264 patients). The sample size ranged from 114 to 21,684. Of the 23 studies we included, 4 were prospective cohort studies and 19 were retrospective studies. The included patients were mainly from the United States, Korea, China, Japan, and Israel. All studies were considered of moderate to high quality, achieving a score of ≥5 based on the NOS.

**TABLE 2:** Characteristics of eligible studies.

Reference	Design	Country or region	Period of study	Diagnostic criteria	Sample size	Patients in each BMI group	Age (years)	Outcome	NOS
Sakr et al. (9)	PCS	24 European countries	2002	Sepsis	1,326	Normal weight: 1,206 Underweight: 120	Normal weight: 58.4 (19.1) Underweight: 52.6 (21)	Hospital stay, ICU LOS, in-hospital mortality	7/9
Arabi et al. (12)	RCS	28 medical centers in Canada, United States, and Saudi Arabia	1996–2008	Septic shock	1,216	Normal weight: 1,020 Underweight: 196	Normal weight: 62.2 (16.8) Underweight: 59.1 (19.2)	Hospital stay, ICU LOS, in-hospital mortality	6/9
Chae et al. (28)	RCS	Korea	2008–2012	Severe sepsis or septic shock	575	Normal weight: 489 Underweight: 86	Normal weight: 64.3 (14.3) Underweight: 63.1 (18.9)	Hospital stay, ICU LOS, in-hospital mortality	7/9
Kuperman et al. (13)	RCS	United States	2007–2010	Sepsis	310	Normal weight: 261 Underweight: 49	Normal weight: 61.2 (19.2) Underweight: 60.3 (22.9)	Hospital stay, in-hospital mortality	6/9
Gaulton et al. (14)	RCS	United States	2005–2007	Severe sepsis	582	Normal weight: 480 Underweight: 102	Normal weight: 57 (44–70) Underweight: 57.5 (43–69)	Hospital stay, 1 year mortality, 28-day mortality	6/9
Katayama et al. (15)	RCS	Japan	2011–2016	Sepsis	195	Normal weight: 163 Underweight: 32	Normal weight: 69.6 (12.4) Underweight: 63.5 (17.1)	ICU LOS, 90-day mortality	7/9
Zhou et al. (16)	PCS	China	2015–2017	Sepsis	131	Normal weight: 98 Underweight: 33	Normal weight: 76.2 (12.8) Underweight: 77.9 (13.2)	Hospital stay, ICU LOS, in-hospital mortality, 90-day mortality	7/9
Juarez et al. (17)	RCS	United States	2010–2016	Severe sepsis or septic shock	114	Normal weight: 88 Underweight: 26	Normal weight: 59.83 (17.81) Underweight: 57.54 (18.92)	Hospital stay, ICU LOS, in-hospital mortality	8/9
Li et al. (18)	RCS	China	2001–2012	Sepsis	2,000	Normal weight: 1,726 Underweight: 274	Normal weight: 67.6 (17.5) Underweight: 66.2 (18.5)	Hospital stay, ICU LOS, in-hospital mortality, 28-day mortality, 90-day mortality, 1 year mortality	6/9
Pepper et al. (19)	RCS	United States	2009–2015	Sepsis	21,684	Normal weight: 18,164 Underweight: 3,520	Normal weight: 72 (18.5) Underweight: 70.6 (18.5)	Hospital stay, ICU LOS, in-hospital mortality	7/9
Ayalon et al. (20)	RCS	Israel	2014	Sepsis	418	Normal weight: 230 Underweight: 188	Normal weight: 5.5 (6.9) Underweight: 5.7 (7.8)	ICU LOS, 28-day mortality	7/9
Jagan et al. (21)	RCS	United States	2015–2018	Sepsis	2,673	Normal weight: 2,281 Underweight: 392	Normal weight: 69.9 (20) Underweight: 69 (17.9)	In-hospital mortality	6/9
Lin et al. (22)	RCS	China	2002–2012	Sepsis	2,838	Normal weight: 2,513 Underweight: 325	Normal weight: 67.56 (16.88) Underweight: 67.02 (17.6)	Hospital stay, ICU LOS, in-hospital mortality, 28-day mortality	6/9
Sato et al. (7)	RCS	Japan	2015	Severe sepsis	417	Normal weight: 314 Underweight: 103	Normal weight: 74.1 (14.2) Underweight: 77.6 (13.5)	In-hospital mortality, 28-day mortality	7/9
Tsai et al. (23)	RCS	China	2013–2017	Sepsis	554	Normal weight: 405 Underweight: 149	Normal weight: 68 (14.4) Underweight: 68.5 (15.2)	Hospital stay, ICU LOS, 28-day mortality, 90-day mortality	6/9
Ito et al. (24)	PCS	Japan	2016–2017	Sepsis	835	Normal weight: 612 Underweight: 223	72.6 (12.6)	In-hospital mortality, 28-day mortality	8/9
Li et al. (3)	RCS	China	2008–2019	Sepsis	1,924	Normal weight: 1,756 Underweight: 168	Normal weight: 76.8 (7) Underweight: 76.8 (7.2)	Hospital stay, ICU LOS, in-hospital mortality, 28-day mortality	7/9
Lv et al. (25)	RCS	China	2008–2019	Sepsis	4,697	Normal weight: 4,207 Underweight: 490	Normal weight: 67.2 (19.2) Underweight: 67 (17.6)	Hospital stay, ICU LOS, in-hospital mortality	6/9
Tanikawa et al. (2)	RCS	Japan	2016–2017	Sepsis	845	Normal weight: 622 Underweight: 223	72.6 (12.6)	In-hospital mortality, 28-day mortality	8/9
Ahn et al. (26)	PCS	Korea	2019–2021	Sepsis	2,481	Normal weight: 1,668 Underweight: 813	Normal weight: 72.3 (13.4) Underweight: 74.2 (14.1)	Hospital stay, ICU LOS, in-hospital mortality	8/9
Li et al. (8)	RCS	China	2008–2019	Sepsis	4,639	Normal weight: 4,208 Underweight: 431	Normal weight: 66.3 (17) Underweight: 67.4 (17)	28-day mortality, 1 year mortality	6/9
Shimizu et al. (27)	RCS	Japan	2014–2018	Sepsis	3,067	Normal weight: 2,229 Underweight: 838	Normal weight: 71.4 (13.2) Underweight: 70.8 (15.8)	In-hospital mortality	8/9
Zhang et al. (1)	RCS	China	2008–2019	Sepsis	4,827	Normal weight: 4,344 Underweight: 483	Normal weight: 66.3 (19.3) Underweight: 68.7 (16.4)	28-day mortality, 90-day mortality, 1 year mortality	6/9

LOS, length of stay; ICU, intensive care unit; PCS, prospective cohort study; RCS, retrospective cohort study.

### 3.3 Meta-analysis

#### 3.3.1 In-hospital mortality

**FIGURE 2:**
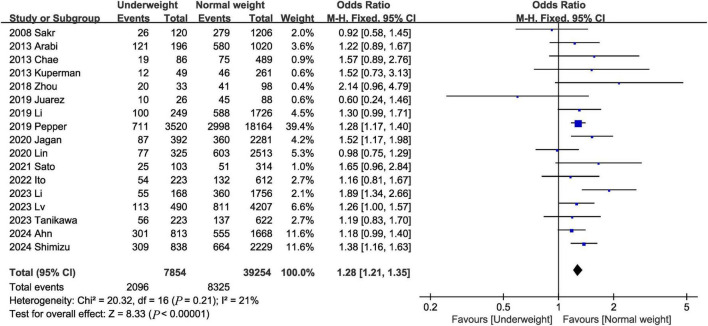
Meta-analysis of in-hospital mortality between underweight and normal weight septic patients.

Seventeen studies (2, 3, 7, 9, 12, 13, 16–19, 21, 22, 24–28) reported data on in-hospital mortality. The combined results of the 17 studies showed that in-hospital mortality was significantly higher in the underweight group than in the normal weight group (OR, 1.28; 95% CI, 1.21, 1.35; heterogeneity: *I*^2^ = 21%, *P* = 0.21) (Figure 2 and Table 3).

**TABLE 3:** Summary of results of meta-analysis.

Outcomes of interest	Studies, *n*	Events for underweight group	Events for normal weight group	MD/OR	95% CI	*P*	*I*^2^ (%)
In-hospital mortality	17	2,096/7,854	8,325/39,254	1.28	1.21, 1.35	<0.00001	21
28-day mortality	11	723/2,644	3,493/17,210	1.54	1.26, 1.88	<0.0001	74
90-day mortality	4	276/696	1,410/5,010	1.46	0.93, 2.30	0.10	74
1-year mortality	4	657/1,265	4,038/10,758	1.78	1.58, 2.00	<0.00001	41
ICU length of stay	13	–	–	−0.06	−0.46, 0.33	0.76	75
Hospital stay	14	–	–	0.05	−0.55, 0.65	0.87	53

#### 3.3.2 28-Day mortality

**FIGURE 3:**
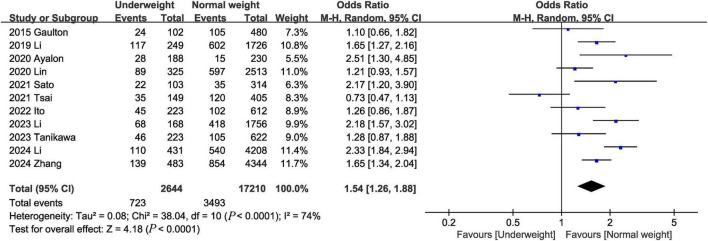
Meta-analysis of 28-day mortality between underweight and normal weight septic patients.

Eleven studies (1–3, 7, 8, 14, 18, 20, 22–24) assessed 28-day mortality. The pooled results showed that being underweight was associated with an increased risk of the 28-day mortality (OR, 1.54; 95% CI, 1.26, 1.88; heterogeneity: *I*^2^ = 74%, *P* < 0.0001) (Figure 3).

#### 3.3.3 90-Day mortality

**FIGURE 4:**
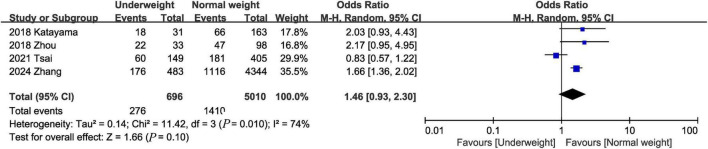
Meta-analysis of 90-day mortality between underweight and normal weight septic patients.

Data from 4 studies (1, 15, 16, 23) of 5,706 patients did not reveal any difference between the underweight and normal weight groups (OR, 1.46; 95% CI, 0.93, 2.30; heterogeneity: *I*^2^ = 74%, *P* = 0.010) (Figure 4) in terms of 90-day mortality.

#### 3.3.4 1-Year mortality

**FIGURE 5:**
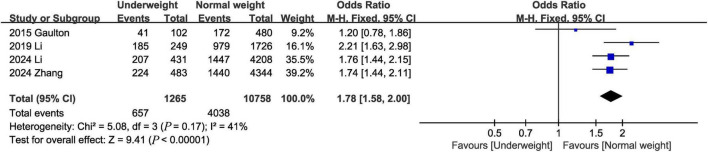
Meta-analysis of 1-year mortality between underweight and normal weight septic patients.

One-year mortality was evaluated in 4 studies (1, 8, 14, 18), and the pooled results showed that the 1-year mortality was higher in the underweight group than in the normal weight group (OR, 1.78; 95% CI, 1.58, 2.00; *P* < 0.00001) (Figure 5).

#### 3.3.5 ICU length of stay

**FIGURE 6:**
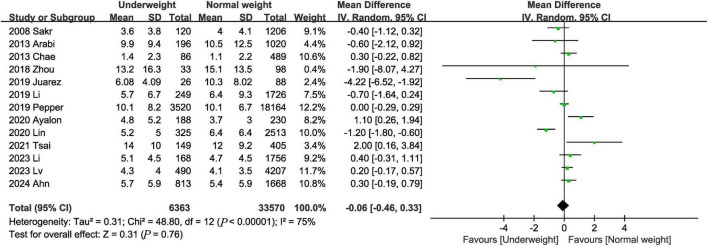
Meta-analysis of ICU length of stay between underweight and normal weight septic patients.

Thirteen studies (3, 9, 12, 16–20, 22, 23, 25, 26, 28) provided information on ICU length of stay. The combined results showed that the underweight group had similar ICU length of stay as the normal weight group (MD, −0.06 days; 95% CI, −0.46, 0.33, *P* = 0.76; *I*^2^ = 75%) (Figure 6).

#### 3.3.6 Hospital stay

**FIGURE 7:**
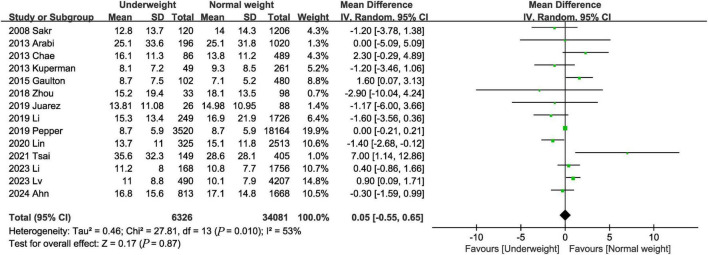
Meta-analysis of hospital stay between underweight and normal weight septic patients.

The hospital stay was reported in 14 trials (3, 9, 12–14, 16–19, 22, 23, 25, 26, 28). The combined results showed that there was no significant difference in length of hospital stay between the underweight group and the normal weight group (MD, 0.05 days; 95% CI, −0.55, 0.65, *P* = 0.87) (Figure 7).

### 3.4 Subgroup analysis

The subgroup analyses were performed based on age and the region where the study was conducted (Table 4). Underweight was associated with an increased risk of in-hospital mortality and 28-day mortality in both the age >70 and ≤70 subgroups. In addition, ICU length of stay and length of stay were not significantly different between the underweight and normal weight groups. When subgroups were analyzed by region, underweight significantly increased in-hospital mortality in both Asian subgroups, European and North American subgroups.

**TABLE 4:** Summary of results from subgroup analyses.

Indicators	Subgrouped by	The number of studies	Effect size	95% CI	*P*	*I*^2^ (%)
In-hospital mortality	Age	–	–	–	–	–
	>70 years	8	1.30	1.21, 1.39	< 0.00001	24
	≤70 years	9	1.22	1.10, 1.36	0.0003	23
	Region					
	Asia	12	1.27	1.17, 1.37	< 0.00001	26
	Europe and North America	5	1.27	1.17, 1.38	< 0.00001	39
28-day mortality	Age					
	>70 years	4	1.64	1.20, 2.24	0.002	57
	≤70 years	7	1.48	1.13, 1.95	0.004	81
Hospital stay	Age	–	–	–	–	–
	>70 years	4	0	−0.21, 0.21	1.00	0
	≤70 years	10	0.12	−1.01, 1.24	0.84	65
	Region					
	Asia	8	0.12	−0.94, 1.18	0.83	67
	Europe and North America	6	0.05	−0.68, 0.78	0.89	21
ICU length of stay	Age	–	–	–	–	–
	>70 years	3	0.05	−0.21, 0.32	0.70	0
	≤70 years	10	−0.14	−0.68, 0.40	0.60	81
	Region					
	Asia	9	0.15	−0.34, 0.64	0.56	76
	Europe and North America	4	−0.76	−1.72, 0.21	0.13	78

### 3.5 Publication bias and sensitivity analysis

**FIGURE 8:**
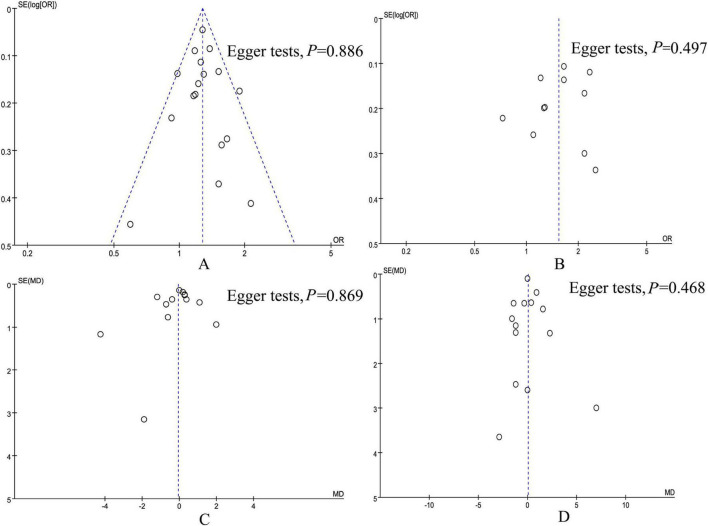
Funnel plot of underweight versus normal weight. **(A)** In-hospital mortality, **(B)** 28-day mortality, **(C)** ICU length of stay, and **(D)** hospital stay.

According to the Egger tests and funnel plots, none of the outcomes (in-hospital mortality, 28-day mortality, ICU length of stay, and hospital stay) demonstrated significant publication bias (Figure 8). Sensitivity analysis showed that no single study affected the overall effect size of the in-hospital mortality, 28-day mortality, 90-day mortality, 1-year mortality, ICU length of stay, and hospital stay.

## 4 Discussion

To the best of our knowledge, this is the largest meta-analysis to evaluate the association between underweight and mortality in patients with sepsis. Data from 23 studies with 58,348 patients showed that while being underweight did not increase length of hospital stay or ICU stay, being underweight was associated with an increased risk of in-hospital mortality, 28-day mortality, and 1-year mortality. Our study has important clinical value as we provide evidence through meta-analysis that underweight is a risk factor for mortality, which may help clinicians detect and intervene early in underweight patients to reduce mortality.

Several previous studies have reported that underweight is associated with poorer clinical outcomes for a variety of diseases (29–31). A retrospective study (29) of 21,019 participants showed that underweight subjects (BMI < 18.5 kg/m^2^) were 1.6 times more likely to be hospitalized within a year than those with a BMI > 18.5 kg/m^2^. In addition, emergency room visits and mortality rates are increased in underweight patients. A meta-analysis by Zhao et al. (30) showed that underweight significantly increased postoperative complications in patients with gastric cancer. In addition, several studies have reported that underweight is associated with increased mortality (31, 32). Wier et al. (33) found that underweight orthopedic surgery patients had 1.2 times and 1.5 times higher rates of in-hospital complications and in-hospital mortality than normal-weight patients, respectively. Studies by Haga et al. (32) have shown that being underweight is a risk factor for death in psychopaths. In 2016, Pepper et al. (34) conducted a meta-analysis of data from three studies and found that being underweight increased the risk of death in patients with sepsis, but the difference was not significant (OR, 1.24; 95% CI, 0.79, 1.95). This may be due to the limited number of studies included. A subsequent updated meta-analysis by Bai et al. (4) included more data (12 studies) and showed that underweight was associated with increased mortality (OR, 1.31; 95% CI, 1.11, 1.54). The association between underweight and mortality was also confirmed by our study. Our results suggest that being underweight not only increases the risk of in-hospital mortality, but also increases the risk of 28-day mortality and 1-year mortality. Similarly, several retrospective studies (35, 36) have shown that underweight is associated with an increased risk of death in patients with sepsis. Danninger et al. (35) showed that underweight significantly increased ICU mortality. Lebovitz et al. (36) found that patients with low BMI (BMI ≤ 19 kg/m^2^) had higher mortality and longer hospital stays compared to patients with sepsis with higher BMI. The mechanism by which underweight is associated with an increased risk of death is unclear and may be related to the following factors. On the one hand, fat tissue has an immune function, and underweight patients have less fat tissue. Therefore, underweight sepsis patients may have weakened immunity (36). On the other hand, patients with sepsis have increased catabolism. In early sepsis, glycolysis is the primary source of energy. As the disease progresses, fat and protein become the main sources of energy. Underweight patients have poor energy reserves and are unable to withstand the effects of catabolism (1, 3, 37). Nutritional therapy is beneficial to the rehabilitation and functional state recovery of patients, and is an important strategy that affects the prognosis of patients. A meta-analysis of 25 studies showed that immunonutritional support reduced mortality in patients with sepsis and reduced length of hospital stay compared to controls (38). In addition, early enteral nutritional support in critically ill patients may provide similar protection as high BMI (36). Given that underweight is associated with a poorer prognosis in patients with sepsis, nutritional support for such patients is warranted to improve outcomes in patients with sepsis.

Some studies suggest that the association between underweight and mortality may vary by age (25, 31, 39). Age is the biggest risk factor for hospitalization (29). Aging results in a redistribution of body composition, including weight loss and decreased bone density (8). Ting et al. (40) found that patients’ risk of malnutrition increased with age. In addition, muscle mass tends to decrease with age (41). As a result, there is a higher proportion of underweight in older patients (31). Previous studies have suggested that weight loss associated with muscle tissue loss (sarcopenia) may be associated with worsening prognosis in sepsis (34). Myostatin is a negative regulator of muscle mass and has been reported to be upregulated in diseases associated with muscle atrophy (42). Kobayashi et al. (43) found that reducing myostatin levels in mice reduced sepsis induced liver dysfunction, acute kidney injury, and neutrophil infiltration into the liver and kidney. Therefore, the effect of underweight on mortality may be influenced by age, which is consistent with our results. The results of our subgroup analysis suggest that the OR values of underweight and mortality are higher in the older subgroup than in the younger subgroup. Similarly, Zhang et al. (1) found that the protective effect of obesity on patients with sepsis becomes more pronounced with age. Given the rising prevalence of sepsis in the elderly population and the potential impact of underweight on patient outcomes, clinicians need to pay more attention to the management of this vulnerable population.

Underweight is more common in Asian populations. Considering that the distribution of BMI in different regions may vary, we conducted a subgroup analysis based on region. The results of subgroup analyses showed that underweight was associated with increased mortality in Asia, Europe, and the Americas. This suggests that the association between underweight and mortality can be generalized to other parts of the world.

Our research has several strengths. First, we conducted a comprehensive search, which helped to enhance the reliability of the study results. In addition, we strictly defined underweight and normal weight according to WHO standards, which helps to ensure the homogeneity between studies and enhance the reliability of this study. Finally, we further confirmed the robustness of the results of this study by sensitivity analysis.

This study has the following limitations. First, most of the studies we included were retrospective studies and were affected by the inherent limitations of retrospective studies. Secondly, the heterogeneity of some outcomes (28-day mortality, ICU length of stay, and hospital stay) was high, and although we performed subgroup analysis by age and region, the source of heterogeneity was still not found. Finally, some results (90-day mortality and 1-year mortality) are based on data pooled from a small number of studies, and more studies are needed to validate these results.

## Conclusion

In conclusion, this meta-analysis, based on currently available and updated evidence, suggests that underweight is associated with increased mortality in patients with sepsis. Considering the impact of underweight on the prognosis of sepsis, it is necessary to design and implement appropriate nutritional support programs to improve the prognosis of underweight patients.

## Data Availability

The original contributions presented in this study are included in this article/supplementary material, further inquiries can be directed to the corresponding authors.
